# Toxicological and biochemical analyses demonstrate no toxic effect of Bt maize on the *Folsomia candida*

**DOI:** 10.1371/journal.pone.0232747

**Published:** 2020-05-06

**Authors:** Zhilei Jiang, Lin Zhou, Baifeng Wang, Daming Wang, Fengci Wu, Junqi Yin, Xinyuan Song

**Affiliations:** 1 Jilin Provincial Key Laboratory of Agricultural Biotechnology, Agro-Biotechnology Research Institute, Jilin Academy of Agricultural Sciences, Changchun, China; 2 Jilin Provincial Key Laboratory of Animal Resource Conservation and Utilization, Northeast Normal University, Changchun, China; University of Texas at Dallas, UNITED STATES

## Abstract

The potential effects of Bt (*Bacillus thuringiensis*) maize on non-target organisms must be conducted before the Bt maize is commercially planted. *Folsomia candida* is one of the non-target organisms of Bt maize, also as an important indicator of soil quality and environmental pollution. In this study, a 90-day *F*. *candida* feeding test were conducted to evaluate the potential effects of two Bt maize lines IE09S034 and BT799 and their non-Bt conventional isolines Zong 31 and Zheng 58. The results show that Bt maize lines had no significant effects on the survival rate, reproduction, adult body length, larval body length, and the activities of acetyl cholinesterase, catalase and superoxide dismutase on the *F*. *candida*. Namely, Bt maize had no toxic effects on the *F*. *candida*.

## Introduction

After Bt cotton has been popularized in China, Bt maize become the most valuable genetically modified (GM) plant for popularization and has an important application prospect. Because Bt maize would release Bt proteins into soil ecosystem through its root exudates [1], pollen, stubble decomposition and straw returning [2] and cause environmental risks, so the safety of Bt maize, especially environmental safety, is focused on worldwide. At present, most studies on the environmental risk assessment of Bt plants use field investigation method to analyze the parameters of soil animal community in field. Due to the complexity of soil ecosystem and the inconsistent operations from test operators, the field survey data are often vulnerable and the repeatability is poor.

In recent years, Laboratory study has become an important means to study the effects of Bt plants on soil animal community [3]. Indicator organism is a key factor for the success of Laboratory study. *F*. *candida*, at the bottom of the food chain, is a worldwide omnivorous soil animal. It is parthenogenesis, and has the characteristics of high reproductive rate and short growth cycle. Moreover, *F*. *candida* is resistance to drought, freeze, hypoxia, but sensitive to poisons. And a large number of *F*. *candida* grow in soil and participate in the degradation and recycling of plant residues. Rusek [4] found that some human behaviors including over-tillage, heavy use of chemical fertilizers, herbicides and pesticides would lead to a significant changes in the population density and structure of *F*. *candida*, so the *F*. *candida* is used as an important indicator of soil fertility and health in farmland environment. Therefore, the International Organization for Standardization proposed *F*. *candida* should be used as a model indicator animal of ecotoxicology to detect environmental pollutants [5]. Because *F*. *candida* is frequently exposed to Bt proteins around Bt plants roots in the soil [6], it has been used in environmental risk assessment of GM plants [3, 7–9].

In this study, using Laboratory study method, a 90-day *F*. *candida* feeding experiment were conducted to evaluate the potential effects of the leaf residues of two Bt maize lines on the fitness of the collembolan *F*. *candida*. We not only evaluated the growth, development and reproduction of *F*. *candida*, but also analyzed the effects of Bt maize on the activities of acetyl cholinesterase, catalase and superoxide dismutase in *F*. *candida*.

## Materials and methods

### Plant materials

Two transgenic Bt maize lines and their corresponding non-transgenic near-isolines were used in the study.

One transgenic Bt maize line was IE09S034 containing Cry1IE gene. Its corresponding non-transgenic near isoline was Zong 31. The two maize materials were both provided by the Institute of Plant Science, Chinese Academy of Agricultural Sciences.

The other transgenic Bt maize was BT799 containing Cry1Ac gene. Its corresponding non-transgenic near-isoline was Zheng 58. They were provided by China Agricultural University.

### Indicator organism

The test *F*. *candida* is a series of FCDK series [10, 11]. The population has been consistently reared in our laboratory for three years.

### Materials preparation

The *F*. *candida* in this experiment was prepared as described in the standard method of ISO [5]. Based on this method, the *F*. *candida* was put into cultural box and cultured in a climate chamber at 20 ±1°C in dark. The relative humidity was close to 100%. Pure Baker’s yeast was used as food and placed on the medium of gypsum: activated carbon = 9:1. Deionized water was added into the cultural box once a week to keep certain moisture. The *F*. *candida* eggs laid within three days are collected together and hatched for feeding experiment.

We collected the leaves at maize maturity stage and dried at 60°C for 12 hours. These leaves were crushed by a crusher and stored in a refrigerator at -20°C for feeding *F*. *candida*.

### Feeding experiment

The 10-12-day old *F*. *candida* was fed with the four maize materials leave substituted for the Baker’s yeast respectively. Each maize material was treated as one feeding treatment, and there were four treatments in total. Each treatment set 30 repeats, one repeat contained 10 *F*. *candida* individuals fed in one culture box. A total of 0.02 g leaf powder and 1 mL distilled water were put into cultural box to feed *F*. *candida* in the beginning and refreshed every two days.

### Stability of Cry proteins of leaf diets

The concentrations of the Cry proteins in the maize leaf diets before and after exposed to *F*. *candida* for 2 days were both evaluated. Three repeats were conducted. The concentration of Cry proteins were analyzed by ELISA using 2–3 mg diet sample.

### The survival and the reproduction rates bioassays

Surveys were conducted on the 10, 20, 30, 40, 50, 60, 70, 80 and 90 days, respectively after *F*. *candida* had been fed with maize leaf powder. The number of adults, eggs and larvae in each culture box was counted (S1 Table). The survival and the reproduction rates were separately calculated.

### The effect of Bt maize on the body length of *F*. *candida*

At the end of the 90-day feeding experiment, three adults and three larvae were randomly taken out from each culture box. The length of these adults and larvae were measured under the stereomicroscope (S2 Table).

### Determination of enzyme activity

At the end of the 90-day feeding experiment, *F*. *candida* were collected using 1.5-mL centrifugal tubes. For each treatment, five centrifugal tubes were prepared and each contained about 0.1 g F. candida. The *F*. *candida* in every tube was ground after 1 mL of normal saline (0.9% NaCl) had been put in, then mixed and centrifuged at 10,000 rpm for 20 minutes. Finally, the supernatant was extracted and used for the detection of enzyme activity.

Acetyl cholinesterase (AChE), catalase (CAT) and superoxide dismutase (SOD) activities were measured with ELISA kits from Nanjing Jiancheng Bioengineering Institute (Nanjing, China).

## Results

### Stability of Cry proteins of leaf diets

According to the results of ELISA measurements, the concentrations of Cry1IE and Cry1Ac in the original leaf diets were 971.76±3.96 and 546.38±2.22 ng/g, respectively. But after a 2-day feeding exposure, the Cry1IE and Cry1Ac contents in the diet residues were significantly decreased to 797.93 ± 9.31 (P< 0.001) and 464.45 ±6.25 ng/g (P <0.001), respectively. No Cry protein was detected in the leaf diets of non-Bt maize plants.

### No effect of Bt maize leaves on the survival rate of *F*. *candida*

**Fig 1:**
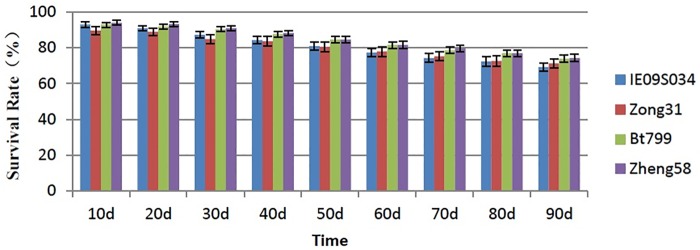
Effects of Bt maize on the survival rate of *Folsomia candida* in the nine investigations. Values are means ± SE, n = 30.

Based on nine samplings during feeding experiment, ANOVA revealed that the survival rate of *F*. *candida* had no significant difference in the leaf-based diets from IE09S034, Zong 31, BT799 and Zheng 58 (P = 0.230) and also there were no significant differences among the treatments in every time (P>0.05) (Fig 1).

### No effect of Bt maize leaves on the reproduction rates of *F*. *candida*

**Fig 2:**
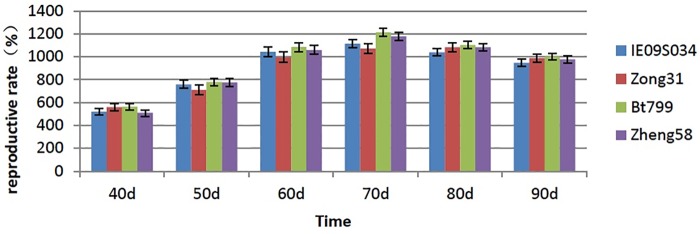
Effects of Bt maize on the reproduction rate of *Folsomia candida* in the six investigations. Values are means ± SE, n = 30.

As the reproductive cycle of *F*. *candida* is about 30 days, we investigated the eggs and larvae of *F*. *candida* once every ten days from the 40th to the 90th days after feeding on maize leaves, namely the investigation was conducted for six times. ANOVA results showed that the reproduction rate of *F*. *candida* had no significant difference in the leaf-based diets from IE09S034, Zong 31, BT799 and Zheng 58 (P = 0.568) and also there were no significant differences among the treatments in every time (P>0.05) (Fig 2).

### No effect of Bt maize on the body length of *F*. *candida*

**Fig 3:**
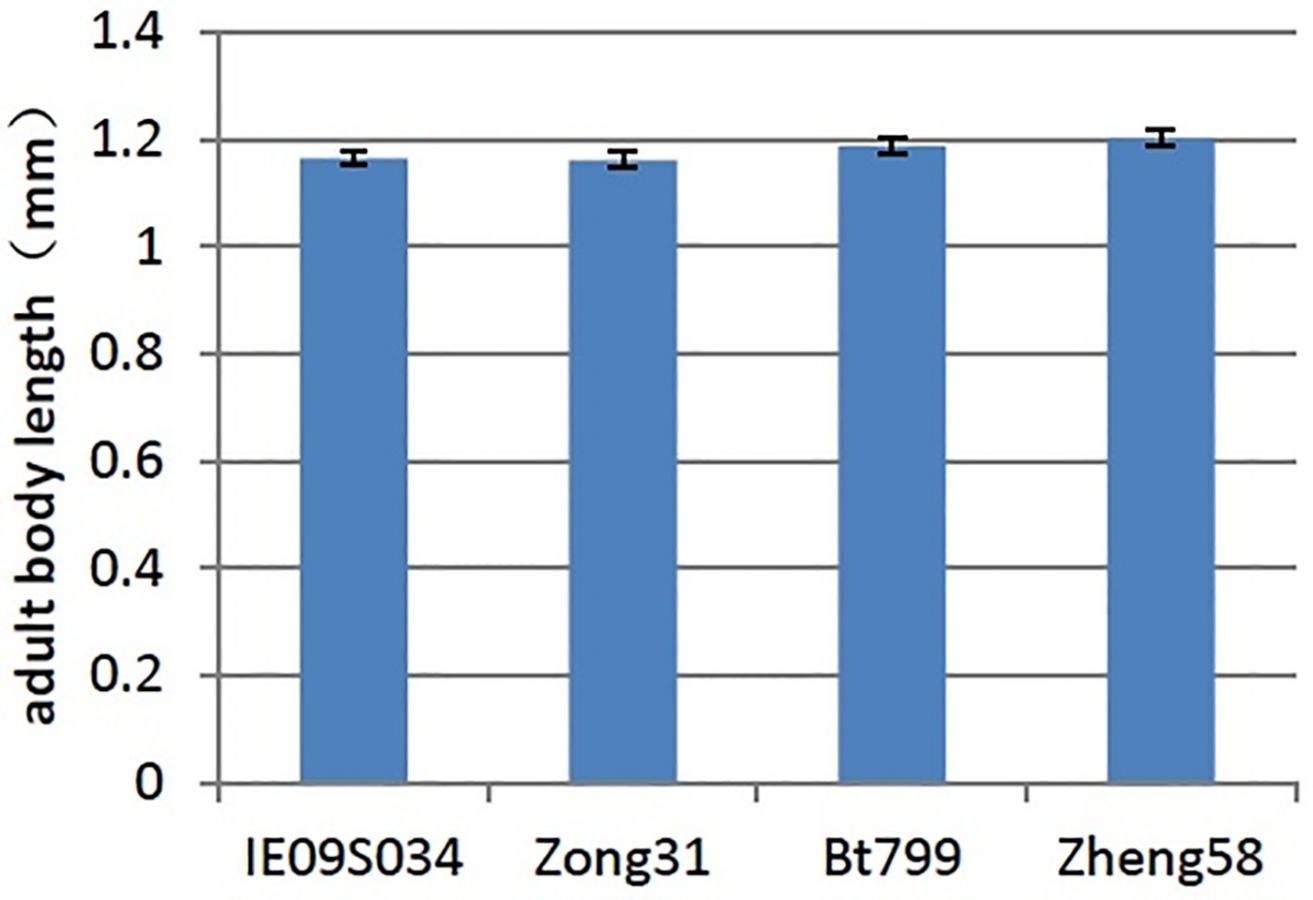
Effects of Bt maize on the adult body length of *Folsomia candida*. Values are means ± SE, n = 30.

**Fig 4:**
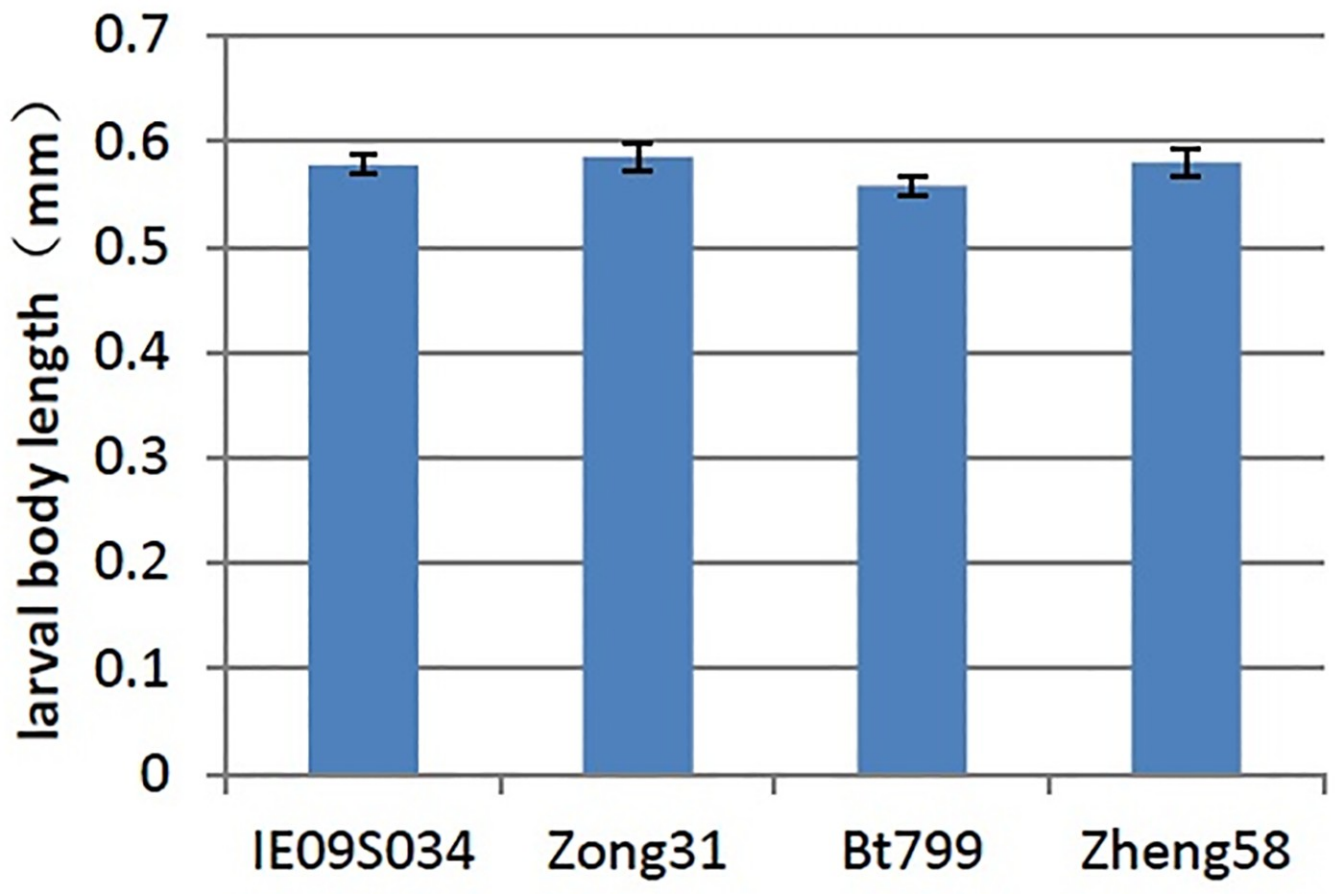
Effects of Bt maize on the larval body length of *Folsomia candida*. Values are means ± SE, n = 30.

After 90 days of culture, the body length of *F*. *candida* was measured. ANOVA results showed, the body length of adult *F*. *candida* had no significant difference in the leaf-based diets from IE09S034, Zong 31, BT799 and Zheng 58 (P = 0.108) (Fig 3); also the body length of larvae *F*. *candida* had no significant difference in the leaf-based diets from IE09S034, Zong 31, BT799 and Zheng 58 (P = 0.342)(Fig 4).

### No effect of Bt maize on enzyme activity of *F*. *candida*

At the end of feeding experiment, three enzyme activities in *F*. *candida* were evaluated. The activity of the three enzymes did not significantly differ in *F*. *candida* that fed on the leaf-based diets from IE09S034, Zong 31, BT799 and Zheng 58 (P>0.05) (Table 1).

**Table 1:** Enzyme activity of *Folsomia candida* feeding on four maize materials.

Enzyme	Variety			
	IE09S034	Zong 31	BT799	Zheng 58
AChE U/mgprot	0.37±0.01a	0.41±0.01a	0.39±0.01a	0.41±0.01a
CAT U/mgprot	72.38±0.41a	71.84±0.61a	71.2±0.38a	73.17±0.45a
SOD U/mgprot	0.71±0.01a	0.69±0.01a	0.69±0.01a	0.72±0.01a

AChE, acetyl cholinesterase; CAT, catalase; SOD, superoxide dismutase.

## Discussion

Usually, *F*. *candida* egg hatch after 7–10 days and the larva grow into the 6th instar (sexual maturity) using 21–24 days, the reproductive cycle of *F*. *candida* is from 28 to 30 days. And the average life span is about 111 days for *F*. *candida*, it spawns once every 10 days, and about 1000 eggs are produced during the whole life [12]. Yuan et al.[8, 13] and Yang et al [3] conducted the studies on feeding *F*. *candida* with Bt proteins all lasted for 28 days. Bai et al.[7] did similar experiment using 35 days. In our study, we did a 90-day *F*. *candida* feeding experiment (covering three reproductive cycles of *F*. *candida*), and during the experiment, investigation were conducted every ten days, so the result in our study were more rigorous and reliable.

To quantify the exposure of *F*. *candida* to Cry protein in the feeding experiments, we used ELISA to confirm the stability of Cry protein in the Bt leaf diet. Our results indicated that >80% of the Cry protein was still detectable in the leaf diet after a 2-day feeding exposure.

The binding of Bt proteins to the specific receptors of target insects is considered to be a key step for the insecticidal action of Bt proteins [14]. Now, the lack of suitable specific receptors in non-target organisms is the main reason that non-target organisms would not to be affected by Bt proteins [15, 16]. Because most non-target organisms lack receptors that interact with Bt proteins, they do not suffer from Bt toxic theoretically. Scientists also conduct a large number of studies about the effects of Bt plant on non-target organisms, and have reveal transgenic Bt plants generally do not have significant adverse effects on the growth and reproduction of non-target organisms. Yu et al. [17] fed *F*. *candida* and *Oppia nitens* with the leaf residues of transgenic Bt cotton and potato, and revealed that transgenic Bt plants had no effects on the reproduction rate and growth of the two experimental animals. Also, the growth and development of *Protaphora armata* around the Bt maize roots was not affected [18]. Sims and Martin [19] concluded that Bt protein did not have a negative effect on the survival and reproduction rates of *F*. *candida* and Xenylla grisea through feeding them with the mixture of CryIAb, CryIAc, CryIIA, CryIIIA and Baker’s yeast. Besides, many non-target animals’ species, such as *F*. *candida* [7, 20], *Eisenia foetida* [20, 21], *Micraspis discolor* [22], have been demonstrated to be not affected by the Bt proteins expressed by transgenic Bt plants.

Many enzymes of insects, such as AChE, CAT, peroxidase (POD) and superoxide dismutase (SOD), response to pesticides and herbicides in farmland [23]. In the study of Yuan et al.[8], both SOD and POD activities of *F*. *candida* were not affected by Cry1Ab and Cry1Ac proteins. Bai et al.[7] also found that there was no significant difference in SOD activity between the *F*. *candida* that was fed by the leaves of Bt transgenic rice and non-Bt rice. Further, Yang et al.[3, 9] fed *F*. *candida* with Cry1Ab and Cry1Ac proteins or transgenic Bt rice, and no significant difference in the studied enzymes activities was found. In this study, we found that there was no significant difference in the activities of AChE, CAT and SOD between the *F*. *candida* fed on the leaves of Bt transgenic and non-Bt maize lines. The result was similar to that of previous studies [3, 7–9].

In this study, we compared the growth and enzymes’ activities of *F*. *candida* fed on Bt maize IE09S034, BT799 and their corresponding non-transgenic maize lines Zong 31, Zheng 58, and no significant differences were found. The results are similar to those of most previous studies, that is, GM plants have no significant adverse effects on the non-target animals.

## Supporting information

S1 Table The number of adults, eggs and larva of *F*. *candida* in each culture box. (XLSX)

S2 Table The body length of adults and larvae of *F*. *candida* in each culture box. (XLSX)
